# Surface Engineering for Mechanical Enhancement of Cell Sheet by Nano-Coatings

**DOI:** 10.1038/s41598-017-04746-x

**Published:** 2017-06-30

**Authors:** Miso Yang, Eunah Kang, Jong wook Shin, Jinkee Hong

**Affiliations:** 1 School of Chemical Engineering and Material Science, Chung-Ang University, 84 Heukseok-ro, Dongjak-gu, Seoul, 06974 Republic of Korea; 2 Department of Internal Medicine, Chung-Ang University College and School of Medicine, Chung-Ang University Hospital, Seoul, Republic of Korea

## Abstract

Cell sheet technology is becoming increasingly popular in tissue engineering and regenerative medicine, due to integrity into versatile organ and manageable cell and tissue type from the bank, and no needs of large volume organ for transplantation. Cell sheets have still a room to resolve the mechanical resistance under load-bearing occasion, easy translocation into organ, and prompt shape modulation for regular application *in vivo*. Herein, a layer-by-layer (LbL) assembly of nanometer scaled film coating method was introduced to inter-planar cell sheet for multilayered cell sheet (M1) and a single cell before sheet formation (M2). Nano-films with collagen and alginate increased mechanical property of cell sheets without altering cell functions, viability, and proliferation. The moduli of triple layered cell sheet (M1) and (M2) were critically enhanced to 109% and 104%, compared to uncoated cell sheet (CON) with mono-layer, while modulus of CON with triple-layers were increased to 43%. LbL assembly to cell sheets offers increased modulus allowing cell sheet engineering to become a potential strategy under load-bearing environment.

## Introduction

Cell-based therapies and tissue engineering are widely used in regenerative medicine^1–3^. Three-dimensional (3-D) biodegradable polymer scaffolds seeded with cells have been used for the regeneration of various host tissues, including bone^4, 5^, skin^6, 7^, cartilage^8, 9^, and heart tissues^10^. However, artificial polymer scaffolds can lead to inflammatory responses and pathological fibrosis. Excessive connective tissue formation and insufficient cell-seeding are considered to be additional limitations to the use of scaffolds in regenerative medicine. Cell sheet engineering was developed as a means to overcome these obstacles. Through this technology, cultured cells are obtained in the form of an intact confluent monolayer sheet including deposited ECM, which can be deployed without the use of any enzymes (e.g., dispase digestion). The harvested cell sheets can be directly transplanted into damaged tissues without the need of mediators such as scaffolds, sutures, or fibrin glue. Furthermore, cell-to-cell junctions and preserved adhesive proteins located underneath individual cell sheets allow the construction of 3-D formations mimicking tissue structure, by stacking 2-D individual cell sheets^11^.

Despite the advantages of cell sheet technology, its widespread use in clinical applications has yet to overcome a series of difficulties. Cell sheets display poor mechanical properties, making them difficult to handle. Plunger-like devices were developed to allow for the transplantation of cell sheets without suffering damage and residual cell losses^12^. Unfortunately, this technique is both costly and time consuming, while its use demands highly skilled and experienced users. Recently, the Okano group presented a scooping device that was designed to allow for easy and rapid transfer of cell sheets from cell culture plates to subcutaneous tissues regardless of manipulator-related variation^13^. Despite the efficiency of the device, their method does not increase the mechanical strength of the cell sheet itself; hence, it cannot be used in applications requiring sheets of high mechanical stability, e.g., for the regeneration of tissues under compressive strain and high pressure.

The materials-integrated cell sheets, thus, have led efforts to fabricate and characterize the hybrid biological system for advanced quantitative understanding^14^. Soft tissue engineering of versatile biopolymer and cell sheets itself were exampled to understand load-bearing tissue-mimicking system as following; anisotropic mechanical properties depending on the micro-patterned cell sheet and oriented extracellular matrix^15^, high mechanical stiffness achieved from cardiomyocyte attachment on poly (N-isopropylacrylamide)^16^, interfacial integration by adhesion of layered collagen^17^, enhanced tensile behavior of mesenchymal stem cells and poly (L-lactide-co-ε-caprolactone) sheet^18^, etc. The combinatory integration with cell sheet and biomaterials seemed to enhance mechanical properties under load-bearing environment, facilitating further sheet translocation and shape modulation *in vivo* application. However, materials employed to cell sheets were not defined to close vicinities of cell membrane in a quantitative manner. This integration with biological materials to cell sheet has a still room to be controlled with defined molecular layer in nano-meter scale, as regarding that oxygen transportation is limited due to the sheet thickness and its permeability after *in vivo* transplantation.

The layer-by-layer (LbL) self-assembly technique is a widespread method of thin film fabrication that is based on alternate immersion into solutions of interactive materials^19^. The LbL assembly not only allows for nanometer-scale control over film thickness, but also can be performed on virtually any kind of substrate^20, 21^, even cell membranes^22^. Through the LbL technique, multi-functional films can be manufactured from diverse materials, such as polymers^23^, proteins^24^, nanoparticles^25^, and therapeutics^26^. For these reasons, LbL multi-layer films have attracted much interest with respect to their potential use in the biomedical field. A large number of studies reporting dedicated biomedical applications of LbL films have already been presented, e.g., nanometer-sized films were fabricated on cell membranes by the LbL method, allowing rapid cell accumulation for construction of 3-D tissues^27^. Moreover, several studies revealed that substrates of cell growth could be coated with LbL multi-layer films, allowing for control over cell fate^28^ or cell functions^29, 30^.

In this study, we developed a simple method for enhancing the mechanical properties of cell sheets by applying LbL-assembled films to them. The LbL multi-layer films were applied either directly on the cell sheet surface (Method 1), or on the surface of cells before the formation of the sheet (Method 2). To the best of our knowledge, this is the first experimental application of the LbL assembly technique to cell sheets as a means of improving their mechanical properties. According to our results, cell sheet surface engineering by LbL coating can enhance the ability of cell sheets to endure compression, without affecting their viability.

## Results and Discussion

### Application of LbL films to cell sheets and characterization of the film components

Collagen is the most abundant protein of the endogenous extracellular matrix (ECM). The isoelectric point of collagen is 9^32^; hence, it possesses a slightly positive charge at physiological conditions (pH range), which is used during LbL film fabrication. Another feature of collagen is its high hydration capacity^33^, which allows it to significantly increase in volume. In specific, collagen type I interacts with α_2_β_1_ integrin membrane receptors with an association constant of 6.7 × 10^4^ (mol/L)^−1^ ^34^, able to physically adsorb on the cellular membrane via noncovalent bond. Alginic acid (AA) is a natural anionic and hydrophilic polysaccharide that displays good biocompatibility in biomedical applications. Its status as a U.S. Food and Drug Administration (FDA)-approved polymer allows it to be one of the most important biomaterials. Furthermore, it presents multitudinous pendant carboxylic acid, which enable sites for heterogeneous mineral nucleation^35^.

**Figure 1:**
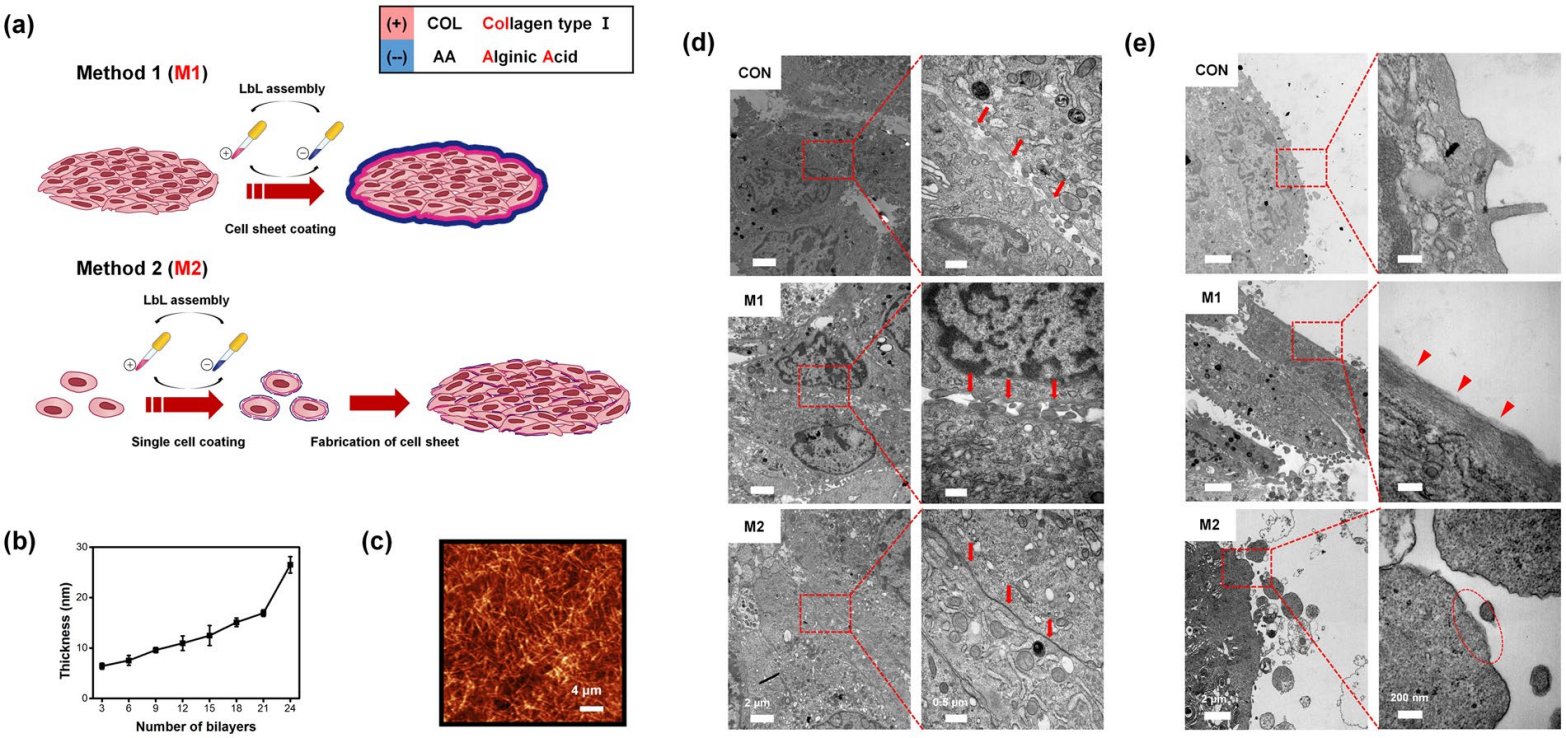
Schematic illustration of two methods for applying LbL films to cell sheets, method 1 (M1) and method 2 (M2) (**a**), the growth curve of the (COL/AA)_*n*_ film as a function of the number of bilayers (**b**), the surface morphology of a film composed of five (COL/AA) bilayers as determined by AFM (**c**). The bar represents 4 μm. TEM images of cell sheet formation (**d**), conformation of films consisting of five (COL/AA) bilayers (**e**). Arrows indicate cell-to-cell junctions, triangles indicate M1 films, and ellipses indicate M2 films. The bars represent 2 μm, 0.5 μm and 200 nm, respectively.

LbL multi-layer films were fabricated by sequential adsorption of COL and AA through electrostatic interactions. Growth of (COL/AA)_*n*_ multi-layer films was found to be a linear function of the number of bilayers (Fig. 1b). This was in agreement with QCM data (Supporting information 1) that demonstrated a linear increase in the absorbed mass. These results showed that (COL/AA)_*n*_ multi-layer films could be successfully constructed through electrostatic binding of COL and AA. We assessed the surface morphology of dried (COL/AA)_5_ multi-layer films by using AFM (Fig. 1c). The image revealed that COL was a dominant component of LbL films and that the films had assembled compactly, which suggested that (COL/AA)_5_ multi-layer films were durable^36^. As a large number of carboxylic acid groups of AA can bind calcium ion of cell growth medium, (COL/AA)_*n*_ multi-layer films has good hardness^37^. AFM analysis measured the RMS roughness of the surface at 5.68 nm.

### Ultrastructure of cell sheets and conformation of (COL/AA) multi-layer films applied to cell sheets

(COL/AA)_5_-coated C2C12 cell sheets were visualized by TEM images to investigate preservation of tight cell-to-cell junctions and new integration between coated (COL/AA)_5_ and cellular membrane at the close vicinity, compared to uncoated cell sheet (CON) (Fig. 1d). Comparison of the distance between cells in CON and cell sheet created by M1 suggested that connections between cells were maintained during cell sheet coating with (COL/AA)_5_ multi-layer film. Cells of M2-created sheet displayed shorter cell-to-cell distances compared to both CON and M1 sheets due to the ability of the cell surface-deposited ECM molecules comprising the films to contribute to cell-cell interactions in ways similar to natural ECM components^27, 38^.

TEM images of the cells located at the edge of the cell sheets showed an absence of film coating in CON sheet and the existence of (COL/AA)_5_ multi-layer films in M1- and M2-created sheets (Fig. 1e). As shown in high-magnification TEM images, the cell membranes of CON sheet display a bare surface, whereas in M1-created sheet, a (COL/AA)_5_ film (red triangle) completely envelops cell membranes. The thickness of the film is about 54 nm (red triangle), i.e., thicker than those that can be fabricated using wafers due to sheet roughness-related limitations. In M2-created sheets, the (COL/AA)_5_ film only partially covers cell membranes (red ellipse), because of film damage or detachment during cell proliferation.

### Viability and proliferation of single cell coated with (COL/AA)_*n*_ multi-layer films

**Figure 2:**
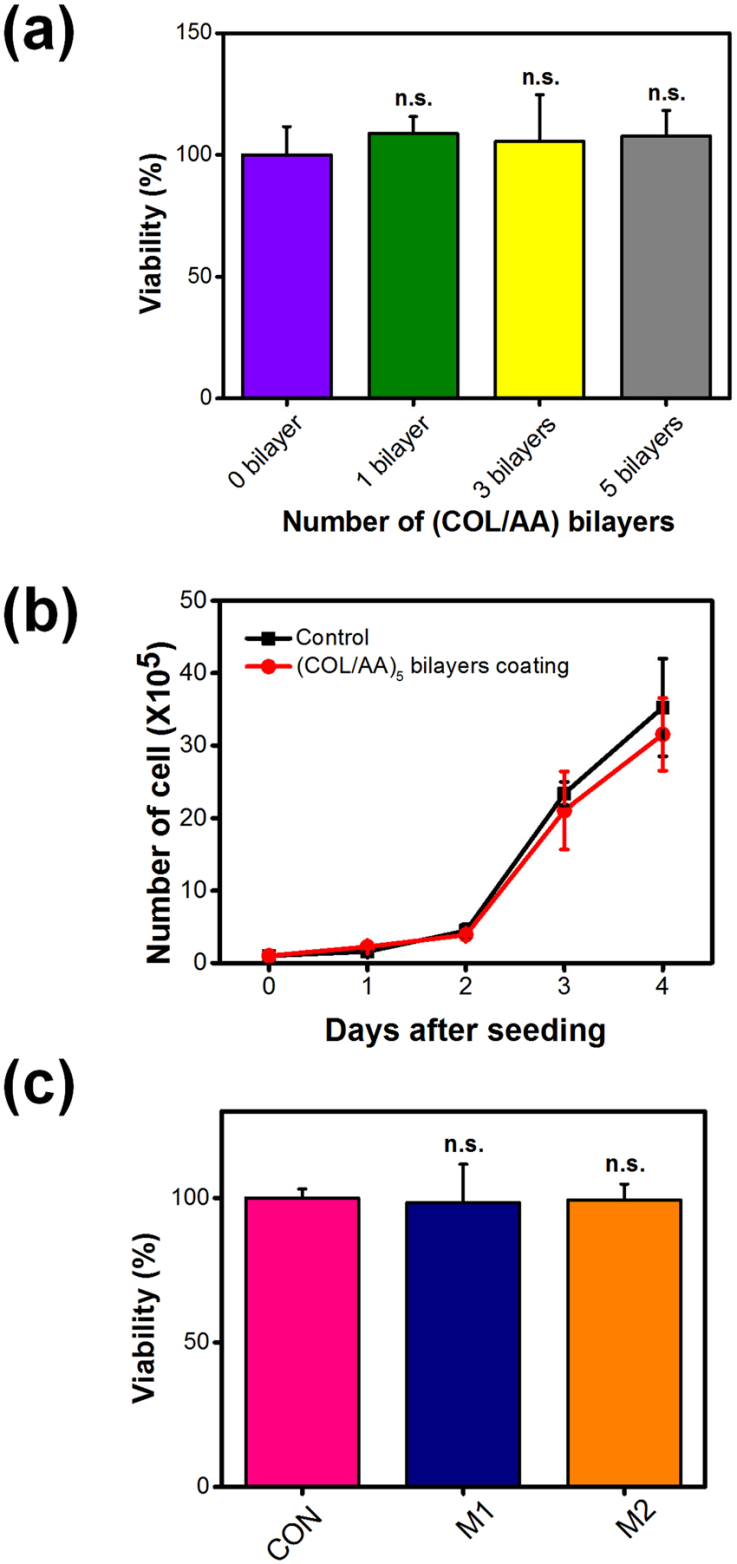
C2C12 single cell viability as a function of the number of (COL/AA) bilayers comprising the multi-layer films that are fabricated on the cell surfaces (**a**), proliferation of C2C12 cells coated in films consisting of five (COL/AA) bilayers, as well as of uncoated cells, for 4 days after seeding (**b**), and viability of CON cell sheets and sheets coated in (COL/AA)_*5*_ multi-layer films fabricated through the two different application methods (**c**). Statistical analysis performed by Tukey’s method and n.s., not significant; versus control.

Viability test results indicated that (COL/AA)_*n*_ multi-layer films coating the membrane surface of C2C12 cells were not cytotoxic (Fig. 2a). The cytotoxicity of polymers that are deposited onto cell membranes depends on characteristics such as molecular weight and charge density^39–41^. As cell membrane mobility is very important to cell viability, damage to the membrane leads to high cytotoxicity^41^. COL has a small positive charge at the physiological conditions maintained during film fabrication and binds to cell surface receptors, allowing it to be coated onto the cell surface without damaging the membrane. Furthermore, both film components, i.e., COL, which is a natural ECM protein, and AA, which is an FDA-approved polymer, are biocompatible materials. Finally, the ability of COL to absorb large amounts of water in aquatic environments allows (COL/AA)_*n*_ multi-layer films to transfer soluble factors that are needed for cell growth, including oxygen and several nutrients.

For reasons mentioned above, proliferation of C2C12 cells is not hindered by the (COL/AA)_*n*_ multi-layer film coating. As seen in Fig. 2b, there was no significant difference in the number of cells between cultures of uncoated cells and cultures of cells coated with a (COL/AA)_5_ multi-layer film, after 4 days in the adopted growth conditions. Figure 1e shows that the force applied during cell division is strong enough to split the (COL/AA)_5_ film coating layered on the surface of the cell membrane. These data suggest that (COL/AA)_*n*_ multi-layer films coated on cells do not affect cell functions.

### Viability of cell sheets prepared with various methods

Viability tests of CON cell sheets and coated cell sheets fabricated through M1 or M2 demonstrated that the application of (COL/AA)_*5*_ multi-layer films to cell sheets has no harmful effects (Fig. 2c). Viability of cell sheet with (COL/AA)_5_ film coating was not critically changed after the LbL procedure, compared to that of the control cell sheet within 5% of viability, as shown in Fig. 2c. Considering the total incubation time of 100 min to perform the multiple LbL coating of (COL/AA)_5_, the preserved viability of cell sheet (M1) indicate that cell sheet were harvested without critical damage and LbL multi-layer film coating onto cell sheet does not affect cell viability. The results corresponded with the one obtained from the single cell viability tests, serving as an additional indication that cell sheets coated with (COL/AA)_*n*_ multi-layer films have the potential to be used in cell therapy.

### Assessment of the mechanical properties of cell sheets

**Figure 3:**
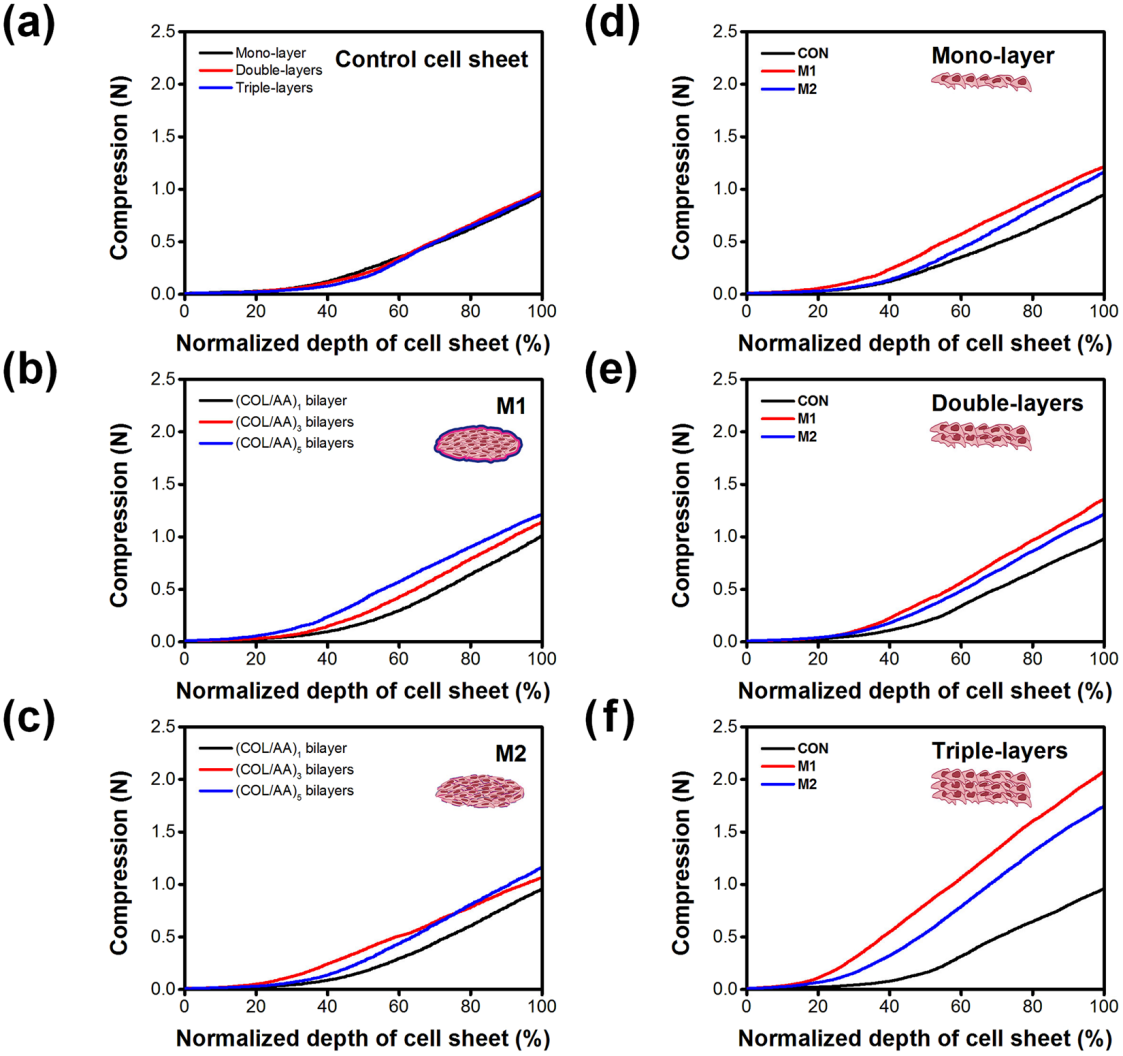
Mechanical properties of control cell sheets composed of one, two, or three cell layers (**a**), the effects of applying (COL/AA)_*n*_ multi-layer films consisting of a diverse number of bilayers on mono-layer cell sheets via M1 (**b**) or via M2 (**c**), comparison between CON, M1, and M2 with respect to the mechanical properties of mono- (**d**), double- (**e**), and triple-layer cell sheets (**f**).

Layered cell sheets with different methods of LbL multi-layer films coating introduction were mechanically characterized by indentation. Identical load-displacement curves were resulted at the high ratio of specimen to indentor diameter (>4)^14^. Load-displacement curve was plotted by applied compression force against the normalized depth of the cell sheets. Curve data of uncoated control cell sheets with one to three cell layers were not significantly different, showing independence of load-displacement behavior as the number of layers (Fig. 3a). The loading–displacement curve of cell sheets showed more nonlinear behavior, which is different from general agarose hydrogels and the applied force to deflect cell sheets is need less than that of hydrogel^42^. The modulus of the nonlinear viscoelastic mechanical behavior of cell sheet were calibrated at the major linear gradient period. The moduli of control cell sheets were varied from 4.2 ± 0.3 to 6.0 ± 0.1 kPa as the number of cell layers (Table 1). This can be explained by that cell sheets as one to three layers require the similar range of compression force to reach same normalized displacement.

**Table 1:** Stiffness and modulus of layered cell sheets prepared with CON, M1 and M2.

		Stiffness (N/m)	Modulus (kPa)
Mono layer	CON	12.5 ± 0.9	4.2 ± 0.3
	M1	17.8 ± 0.8	5.9 ± 0.3
	M2	17.0 ± 0.9	5.7 ± 0.3
Double layers	CON	16.7 ± 0.9	5.6 ± 0.3
	M1	20.1 ± 0.9	6.7 ± 0.3
	M2	19.4 ± 0.4	6.5 ± 0.1
Triple layers	CON	17.9 ± 0.4	6.0 ± 0.1
	M1	26.3 ± 0.6	8.8 ± 0.2
	M2	25.9 ± 0.2	8.6 ± 0.1

Polyelectrolyte films (COL/AA) of 1, 3, and 5 bilayers were introduced to mono-layer cell sheets by M1 and M2, respectively (Fig. 3b and c, respectively). The gradient indicating stiffness in load-displacement curve was gradually increased in both M1 and M2-performed cell sheets as the number (n) of bilayers comprising the (COL/AA)_*n*_ multi-layer films were increased (Supporting information 3a and 3b). Calibrated moduli were 5.9 ± 0.3 and 5.7 ± 0.3 for of monolayer cell sheets performed (COL/AA)_5_ bilayer coating by M1 and M2 methods, respectively (Table 1 and Fig. 3d). Significant difference of modulus in (COL/AA)_5_ coated monolayer cell sheet was not observed, probably because the indentation resolution for monolayer cell sheet is not reached to measure the difference of mechanical properties. In the same manner, compression vs. displacement were measured for double layered and triple layered cell sheets coated with (COL/AA)_5_ bilayers (Fig. 3e, Fig. 3f). The gradient in load-displacement curve were increased in both double layered and triple layered cells sheets carried with M1 and M2, compared to those of CON, respectively. Higher gradient indicated stiffer mechanical properties of cell sheets which can be facilitated for translocation and load-bearing potentials. Calibrated moduli were 6.7 ± 0.3 and 8.8 ± 0.2 kPa for (COL/AA)_5_ coated double layered and triple layered cell sheets (M1), respectively (Table 1). And, (COL/AA)_5_ coated double layered and triple layered cell sheets (M2) presented 6.5 ± 0.1 and 8.6 ± 0.1 kPa, respectively (Table 1). The moduli of triple layered cell sheet (M1) and (M2) were critically enhanced to 109% and 104%, compared to CON with mono-layer, while modulus of CON with triple-layers were increased to 43%. This result clearly presented that the LbL coating with (COL/AA) on cell sheet is major factor to enhance mechanical moduli and the stiffness, rather than the number of layered cell sheet. As expected, the cell sheet itself is limited by adding the number of cell layers in order to enhance mechanical properties and to satisfy requirement of translocation and load bearing environment *in vivo* applications. Incorporation of LbL polymeric layer into cell sheet might be effective methods to increase modulus without disintegration at the interface between cell membrane and polymeric layers, as corresponding to the TEM images.

The coated (COL/AA)_5_ multi-layer films prepared on flat substrates measured by QCM were 220 μg/cm^2^ in weight and 7 nm in thickness. It was notable that the minimal amount of polymer was introduced into cell sheet and satisfied the enhancement of mechanical properties, together with preservation of biological cell function and viability. The choice of material might be critical since the collagen in our study was chosen for adhesion with α_2_β_1_ integrin membrane and electrostatic interaction with alginic acid. This is good agreement with reported studies that LbL assembled single cells with adhesion protein, fibronectin and counter part of polyelectrolytes, gelatin were resistant to physical stress due to the nanometer-scaled meshwork around cellular membrane^41, 43^. Our study is significant in that minimal amount of LbL electrostatic polymeric coating enhanced the mechanical properties, which can further be supportive for translocation and for mechanical demand under load-bearing environment.

Modulus and stiffness of cell sheet performed by M1 was always slightly higher than those carried by M2 (Supporting information 3c and 3d). It was interested in that single cells coated with M2 (LbL coating before sheet formation) generated enhanced moduluscompared to uncoated control cell sheet. Time was allowed for proliferation and ECM secretion after LbL coated individual cells were seeded. The addition of small amount of collagen to adhere the cellular membrane and alginic acid to interact electrostatically with collagen and further secreted ECM might be contributed to the enhanced mechanical properties. Supporting this suggestion, polymeric LbL multi-layer films coating of multilayered cell sheet carried by M1 were introduced to the interface between prepared monolayer cell sheets, allowing only the time to adhere large area of the cell sheet. The results of cell sheet carried by M1 and M2 suggested that interfacial adhesion between cell sheets is critical to augment mechanical properties. The developed method to fabricate cell sheet, combining LbL method provided improved mechanical stiffness with fine tuning. As the number of cell sheet layer and applied LbL methods, mechanical properties of cell sheet were given as compressive stiffness. The stiffnesses of triple layered cell sheet were between 33–41 kPa (Table 2). Considering evaluated mechanical stiffness of normal cell-cell interaction (2–5 kPa)^44^ and skeletal muscle engineered tissue (18–25 kPa)^45^, mechanical stiffness of triple layered cell-sheet of myoblastic C2C12 also satisfied the required stiffness for potentially utilizing multilayered cardiac tissue, as well as easy manipulation. High cell viability of layered cell sheets also proved successful physiological connection and constructed network between the cells and sheets.

**Table 2:** Compressive stress of multilayers.

	Compressive stress (mN/mm^2^ or kPa)		
	Mono-layer	Double-layers	Triple-layers
CON	33.7	34.7	33.9
M1	36.6	40.6	43.2
M2	34	37.9	41.1

The cell sheets with/without LbL coating showed both nonlinear mechanical responses, indicating that LbL coating preserved viscoelastic feature of soft biological tissue. In general, soft tissue models (ex. alginate hydrogel) with viscoelastic properties has estimated nonlinear mechanical response to validate biological soft tissues with a varied ranges of stiffness^14^. Considering that relatively linear mechanical response of 2% alginate hydrogel^42^, it is notable that our LbL coating minimizes the alteration of nonlinear mechanical response, whereas the stiffness of cell sheets was enhanced as the number of LbL coating. It is suggested that our LbL coating is suitable for fine tuning of mechanical stiffness, mimicking soft biological tissue.

## Conclusions

LbL assembly with ECM protein was introduced to the single cells and cell sheets for the purpose of improving mechanical properties. LbL assembled cell sheets demonstrated enhanced modulus, without altering cell function or viability. LbL multi-layer films with collagen interacting with cellular membrane and alginic acid taking charge counterpart for electrostatic interaction, take account the augmented mechanical properties of cell sheet. Only five number of LbL multi-layer films presented that the moduli of triple layered cell sheet (M1) and (M2) were critically enhanced to 109% and 104%, compared to CON with mono-layer, while modulus of CON with triple-layers were increased to 43%. Minimally incorporated natural polymer into cell sheet granted the fine tuning of mechanical properties by controlling nanometer-scaled thickness and amount via LbL fashion. Our quantitative study showed evidently that LbL multi-layer films of natural polymer into cell sheet is the positive tool to tune fine mechanical properties, which might be further integrate into organ with diverse mechanical modulus, translocate simply into organ and design irregularly figured shape modulation.

## Methods

### Materials

Rat-tail tendon collagen type I (COL) (Santa Cruz Biotechnology, Dallas, TX, USA) and alginic acid (AA) (Sigma Aldrich, St. Louis, MD, USA) were used without further purification. Dulbecco’s modified Eagle medium (DMEM), fetal bovine serum (FBS), antibiotics, and trypsin were obtained from Thermo-Fischer Scientific (Waltham, MA, USA).

### Fabrication and characterization of LbL films on flat substrates

COL and AA were dissolved in DMEM at a concentration of 1 mg/mL. The substrates were treated with O_2_ plasma for 2 min prior to use. Further, they were alternately dipped in each solution for 10 min, and washed with DMEM for 2, 1, and 1 min. Film thickness was measured by a Dektak 150 profilometer (Veeco, Oysterbay, NY, USA). The amount of adsorbed film was determined using a QCM200 quartz crystal microbalance (Stanford Research Systems, Sunnyvale, CA, USA). Before use, the QCM200 electrodes were treated with piranha solution (made with a 3:2 ratio of concentrated H_2_SO_4_ and 30% H_2_O_2_) for 5 min, rinsed with deionized water, and finally, dried with N_2_ gas to clean the surface. Film mass was calculated using the Sauerbrey equation, after measuring the change in frequency of the QCM crystal. Film surface morphology and root mean square (RMS) roughness were analyzed by non-contact atomic force microscopy (AFM) using an NX-10 microscope (Park Systems, Suwon, Korea).

### C2C12 cell culture, preparation, and three-dimensional stacking of cell sheets

A mouse embryonic myoblastic cell line, C2C12, was obtained from American Type Culture Collection (ATCC). Cells were grown in DMEM supplemented with 10% FBS and 1% antibiotics at 37 °C in a humidified 5% CO_2_ incubator. After trypsinization, cells were harvested and seeded in 35-mm cell culture dishes at a density of 2 × 10^6^ cells/dish. After incubation for 10 days in the aforementioned growth conditions, cultured cells had reached the desired level of confluence and formed a monolayer. To obtain first layer of cell sheet, the circled line on the edge of cell culture dish were drawn with a point of cell scraper. From the slit of monolayer on the edge, cell sheet was carefully laid off with aid of cell scraper. Whole cell sheet was obtained without any rift. To obtain a 3-D sheet, a cell sheet was transferred onto another cell sheet. The two overlapped monolayers were incubated at the same growth conditions for 30 minutes, to allow for cells from the two sheets to adhere to each other via cell-to-cell junctions. This process was repeated to obtain a triple-layered cell sheet.

### Application of LbL films to cell sheets

All handling was carried out in a clean bench under sterile conditions. COL and AA were dissolved in growth medium at a concentration of 1 mg/mL. The prepared solutions were sterilized using 0.2 μm syringe filters. There are two methods of applying LbL films to cell sheets (Fig. 1a). The first method (M1) is direct application of the multi-layer film on the cell sheet surface. The harvested mono- or multi-layer cell sheets were dipped in the COL solution inside an incubator for 10 min, and washed to physically remove weakly adsorbed COL. Further, they were immersed in the AA solution inside an incubator for 10 min and washed again. The process was repeated several times, to produce the desired number of film layers.

The second method (M2) is coating the cells with multi-layer films prior to the construction of the cell sheet. First, LbL multi-layer films were deposited on cell membrane surfaces. Specifically, after trypsin treatment, the cell pellet was collected by centrifugation and resuspended in COL solution via gentle pipetting. After centrifugation, the supernatant was discarded, and the cell pellet was washed twice with growth medium to remove unbound COL. The process was repeated until the desired numbers of alternating COL and AA layers of film had been adsorbed. The coated cells were seeded in cell culture dishes to produce the sheets.

### Transmission electron microscopy (TEM) images

C2C12 cell sheets were fixed in Karnovsky’s fixative solution for 2 h at 4 °C. They were washed with 0.05 mol/L sodium cacodylate. Post-fixation was performed in 1% OsO4 for 2 h at 4 °C. After being washed with deionized water, the sheets were stained with 0.5% uranyl acetate. They were dehydrated using a graded series of ethanol. Finally, they were embedded in epoxy resin by drying in an oven at 70 °C for 24 h. An EM UC7 ultramicrotome (Leica Camera, Wetzlar, Germany) was used to obtain thin sections, which were mounted on copper grids and analyzed with a JEM1010 microscope (JEOL, Tokyo, Japan) at RT.

### Analyses of the functions of cells and cell sheets

The viability of single cells and cell sheets was confirmed by the MTT assay. For single cell viability tests, C2C12 cells were seeded in 96-well culture plates at a density of 1 × 10^4^/cm^2^. After 24 h, the culture medium was replaced with a 5 mg/mL MTT solution, and the plate was incubated at 37 °C for 3 h. The formazan crystals that formed during the process were dissolved in dimethyl sulfoxide (DMSO) in a 37 °C incubator for 30 min. After brief shaking, absorbance at 540 nm was measured by a SpectraMax i3x plate reader (Molecular Devices, Sunnyvale, CA, USA). Similarly, viability tests of cell sheets were performed by applying 5 mg/mL MTT and following the aforementioned steps. Proliferation of C2C12 cells was assessed by cell counting. C2C12 were seeded in 6-well culture plates at a density of 1 × 10^4^/cm^2^. Cells were harvested by trypsin treatment, counted with a hemocytometer, and reseeded in culture plates. The counting procedure was performed every day after seeding.

### Evaluation of the mechanical properties of cell sheets

Cell sheets to be used for tests of mechanical properties were separated from the growth medium just before measurement. The strength of the cell sheets was assessed with a compression analyzer designed for compressive strength tests of small-sized smooth materials (Supporting information 2). The indentor was equipped with a 4.9 N load cell (resolution of 0.98 mN) and a 3 mm diameter flat ended cylindrical stainless steel indentation probe. Force was applied to cell sheets vertically and the rate of force measurement was 10 μm/sec. We considered the depth under 4.9 mN as the initial depth of the cell sheets (L_0_), and the depth when the measuring tip reached the plate dish as the total depth (L_t_) of the sheets. The length of the cell sheets, L, was calculated as the difference between L_t_ and L_0_, and each measured depth was normalized to L. The mechanical properties of the cell sheets were obtained from the applied compression force (N), which was measured at each point of the cell sheet depth. All values were the average of three measurements.

The compressive moduli of soft tissue have been directly calibrated using stress-strain curve. In this study, load-displacement data from indentation measurement were used to obtain moduli by mathematical model by Oliver and Pharr^31^. Effective modulus, E^*^, was

1 $$\frac{1}{{E}^{\ast }}=\frac{1-{\upsilon }_{s}^{2}}{{E}_{s}}+\frac{1-{\upsilon }_{i}^{2}}{{E}_{i}}$$

where E and υ are Young’s modulus and Poisson’s ratio, respectively, and the subscripts s and i present specimen and indentor. The each term indicate elastic displacement resulted from a sample and indentor. However, as indentor was rigid compared to the soft specimen (stainless steel vs. cell sheet), contribution from second term can be ignored.

Most widely used mathematical model of indentation for soft tissue can be expressed as following,

2 $${E}^{\ast }=\frac{1}{\beta }\times \frac{dP}{dw}\times \frac{1}{2}\times \frac{\sqrt{\pi }}{A}=\frac{S}{2a}$$

where β is a geometric constant such that β = 1 for flat ended indentation probe and *dP/dw* is the stiffness (S) at instantaneous displacement (*w*) and load (*P*). Derivatives (*dP/dw*) of incorporation with load and instantaneous displacement is used to considered for non-linearity of soft tissue. Measured stiffness at each measurement was accounted to obtain calibrated effective moduli.

## Electronic supplementary material

Supporting information
