# Supplementary material for: Surface Engineering for Mechanical Enhancement of Cell Sheet by Nano-Coatings
Source: Sci Rep. 2017 Jun 30;7:4464. doi: 10.1038/s41598-017-04746-x (PMC5493676; doi:10.1038/s41598-017-04746-x)
Supplement: Supplementary file 1 — Supporting information [file 41598_2017_4746_MOESM1_ESM.doc]

Supporting Information for:

**Surface Engineering for Mechanical Enhancement of Cell Sheet by Nano-Coatings**

Miso Yang^1^, Eunah Kang^1,*^, Jong wook Shin^2,*^& Jinkee Hong^1,*^

1. **The mass growth curve of the (COL/AA)*_n_* film**
2. **Photographs of the analyzer used to assess the mechanical properties**
3. **Stiffness and Modulus of C2C12 cell sheets**


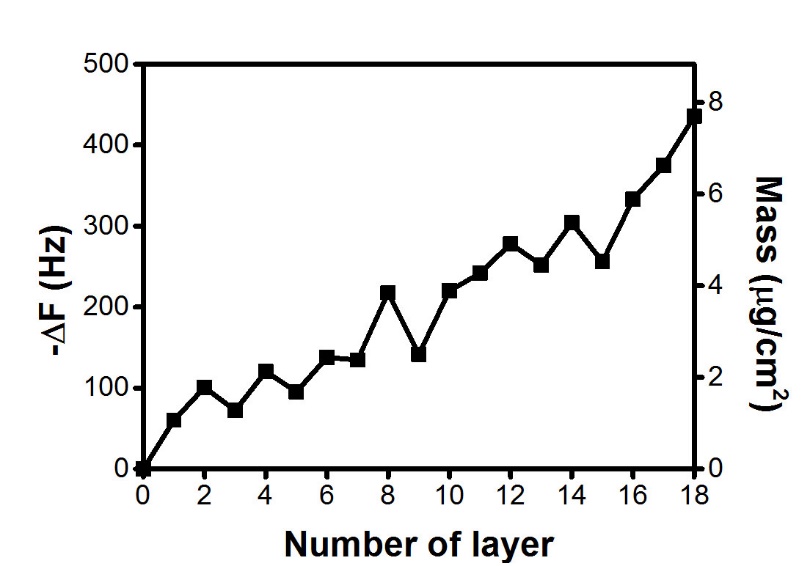


**Fig S1.** Frequency changes (-ΔF) in the QCM are converted into changes in mass resulting from deposition of (COL/AA)*_n_* films. The QCM plot showed fluctuation in patches, whereas some odd layer numbers decreased as a function of depositions, owing to partial detachment of adsorbed COL resulting from its slightly positive charge at the conditions under which films are fabricated. It should also be mentioned that the frequency change in QCM depends not only on the deposited mass, but also on the hydration and stiffness of the materials.


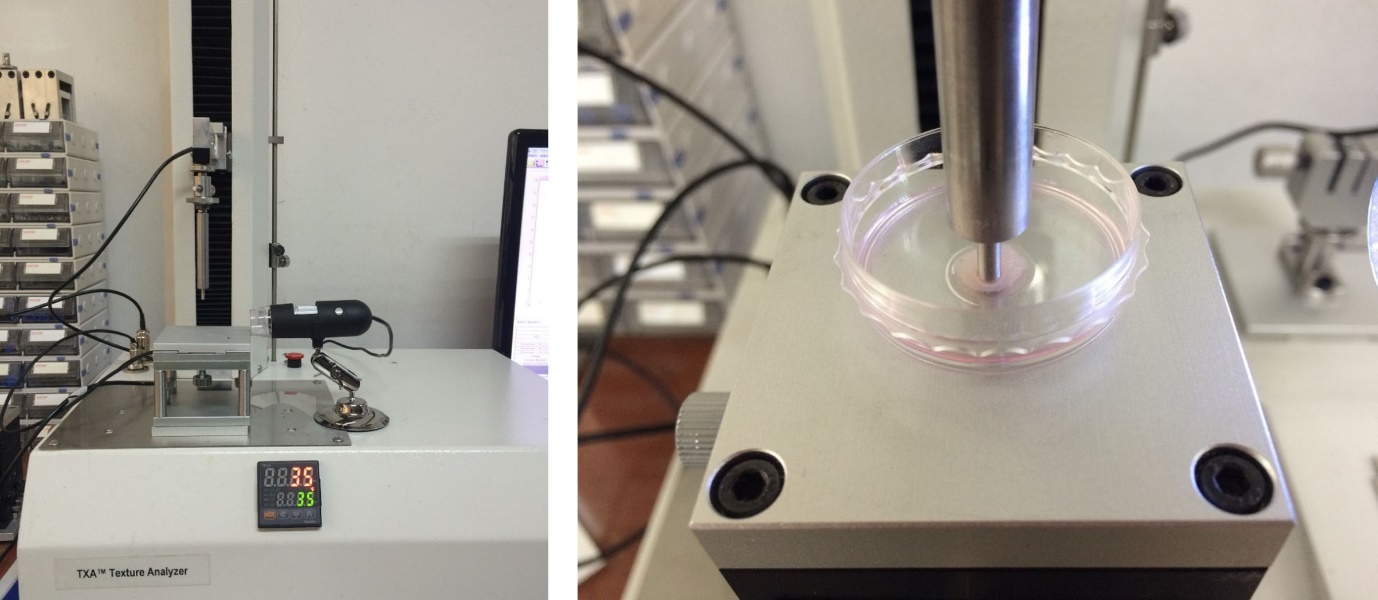


**Fig S2.** Photographs of the analyzer used to assess the mechanical properties of cell sheets. In the right picture, the measuring tip is in contact with a cell sheet during a compressive strength measurement.


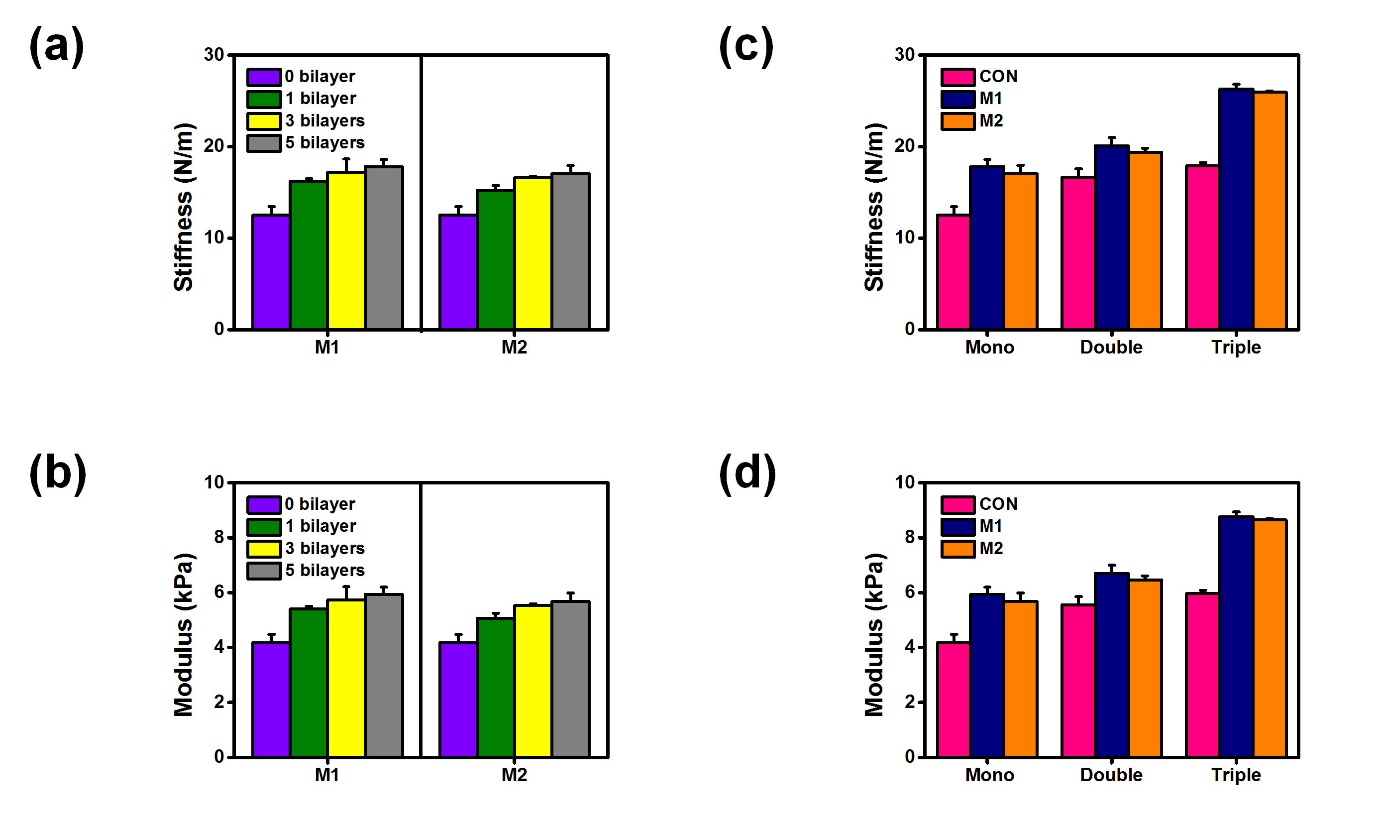


**Fig S3.** Stiffness (a) and modulus (b) of mono-layer cell sheets in terms of applying (COL/AA)*_n_* multi-layer films. Increasing the number of bilayers of (COL/AA) film led to an increase stiffness and modulus of M1 and M2. Comparison of stiffness (C) and modulus (d) between triple-layers cell sheets of CON, M1, and M2.
